# A genome-wide association study of production traits in a commercial population of Large White pigs: evidence of haplotypes affecting meat quality

**DOI:** 10.1186/1297-9686-46-12

**Published:** 2014-02-14

**Authors:** Marie-Pierre Sanchez, Thierry Tribout, Nathalie Iannuccelli, Marcel Bouffaud, Bertrand Servin, Amabel Tenghe, Patrice Dehais, Nelly Muller, Maria Pilar Del Schneider, Marie-José Mercat, Claire Rogel-Gaillard, Denis Milan, Jean-Pierre Bidanel, Hélène Gilbert

**Affiliations:** 1 INRA, UMR1313 Génétique Animale et Biologie Intégrative, F-78350 Jouy-en-Josas, France; 2 INRA, AgroParisTech, UMR1313 Génétique Animale et Biologie Intégrative, F-78350 Jouy-en-Josas, France; 3 INRA, UMR444 Laboratoire de Génétique Cellulaire, F-31326 Castanet-Tolosan, France; 4 INRA, UE450 Testage Porcs, F-35651 Le Rheu, France; 5 IFIP, Pôle génétique, F-35651 Le Rheu, France

## Abstract

**Background:**

Numerous quantitative trait loci (QTL) have been detected in pigs over the past 20 years using microsatellite markers. However, due to the low density of these markers, the accuracy of QTL location has generally been poor. Since 2009, the dense genome coverage provided by the Illumina PorcineSNP60 BeadChip has made it possible to more accurately map QTL using genome-wide association studies (GWAS). Our objective was to perform high-density GWAS in order to identify genomic regions and corresponding haplotypes associated with production traits in a French Large White population of pigs.

**Methods:**

Animals (385 Large White pigs from 106 sires) were genotyped using the PorcineSNP60 BeadChip and evaluated for 19 traits related to feed intake, growth, carcass composition and meat quality. Of the 64 432 SNPs on the chip, 44 412 were used for GWAS with an animal mixed model that included a regression coefficient for the tested SNPs and a genomic kinship matrix. SNP haplotype effects in QTL regions were then tested for association with phenotypes following phase reconstruction based on the Sscrofa10.2 pig genome assembly.

**Results:**

Twenty-three QTL regions were identified on autosomes and their effects ranged from 0.25 to 0.75 phenotypic standard deviation units for feed intake and feed efficiency (four QTL), carcass (12 QTL) and meat quality traits (seven QTL). The 10 most significant QTL regions had effects on carcass (chromosomes 7, 10, 16, 17 and 18) and meat quality traits (two regions on chromosome 1 and one region on chromosomes 8, 9 and 13). Thirteen of the 23 QTL regions had not been previously described. A haplotype block of 183 kb on chromosome 1 (six SNPs) was identified and displayed three distinct haplotypes with significant (0.0001 < P < 0.03) associations with all evaluated meat quality traits.

**Conclusions:**

GWAS analyses with the PorcineSNP60 BeadChip enabled the detection of 23 QTL regions that affect feed consumption, carcass and meat quality traits in a LW population, of which 13 were novel QTL. The proportionally larger number of QTL found for meat quality traits suggests a specific opportunity for improving these traits in the pig by genomic selection.

## Background

Over the past 20 years, several whole-genome scans using mainly microsatellite markers have revealed quantitative trait loci (QTL) for a large number of traits in pigs. To date, a total of some 8300 QTL for more than 600 overlapping phenotypic traits have been reported in the PigQTLdb (http://www.genome.iastate.edu/cgi-bin/QTLdb/SS/index, March 21, 2013; [1]). However, because of the low density of microsatellite markers, these QTL are generally located with poor accuracy and additional long-term fine-mapping experiments are necessary to refine their positions and determine causative variants (e.g. [2,3]). Moreover, QTL linkage designs have in most cases been based on a limited number of families from crosses between divergent populations [4], resulting in limited mapping accuracy and QTL results that are not directly transferable to commercial populations.

The high-throughput genotyping of single nucleotide polymorphisms (SNPs) has become available for pigs with the Illumina PorcineSNP60 BeadChip [5]. The dense genome coverage provided by this chip makes it possible to exploit the linkage disequilibrium (LD) between SNPs and QTL through genome-wide association studies (GWAS). GWAS have been shown to be more powerful for accurate QTL mapping than linkage-based methods (see for example [6] in livestock). Since the PorcineSNP60 BeadChip has become available, several GWAS have been performed in commercial pig populations and have revealed significant associations for economically important traits such as boar taint [7], reproductive characteristics [8], body composition [9], pathogen susceptibility [10], hematological traits [11], feed efficiency [12,13], and meat quality [14]. GWAS have also been performed in a Large White × Minzhu inter-cross population for meat quality [15] and hematological traits [16]. However, to date results on dense GWAS are not available for other major traits in commercial pig populations such as growth rate.

The objective of this work was to perform a high-density genome-wide association study with the PorcineSNP60 BeadChip in the French Large White (LW) breed in order to map the genomic regions that are associated with growth efficiency, carcass and meat quality traits, and to identify haplotypes that may be suitable for inclusion in selection strategies.

## Methods

### Ethical statement

The animals involved in this study were reared and slaughtered in compliance with national regulations applicable to animal research and commercial slaughtering.

### Animals and traits

A total of 385 French LW castrated male pigs from 29 LW nucleus herds were performance-tested at the French national test station in Le Rheu (UETP, Le Rheu, Ille et Vilaine, France). Pigs descended from 106 sires and 313 dams and originated from 335 litters. The piglets entered UETP at 4 to 6 weeks of age and were placed in group pens in a post-weaning unit until they were 10 weeks old. They were then transferred to a fattening unit and placed in group pens for 12 animals equipped with Acema 64 single-place electronic feeders (Acemo, Pontivy, France). All pigs were weighed at the beginning (around 35 kg live weight) and end (around 110 kg live weight) of the performance test. During the test period, they were fed *ad libitum* with pellets composed of cereals and soybean meal containing 9.5 MJ net energy and 156 g crude protein per kg, with a minimum of 0.87 g digestible lysine per MJ of net energy. The average daily gain (ADG), daily feed intake (DFI) and feed conversion ratio (FCR) during the test period were calculated using individual weight measurements and data collected from the electronic feeders. Residual feed intake (RFI) was computed as the difference between DFI and a “theoretical” feed intake predicted from maintenance and production requirements using a phenotypic multiple linear regression method, as described in [17]. When pigs reached 110 kg live weight, they were fasted for a minimum of 16 hours and then transported for about 35 minutes to be slaughtered in a commercial abattoir (Cooperl, Montfort-sur-Meu).

Carcass weight and length were measured shortly after slaughter. Carcass length (CL) was determined from the atlas to the anterior edge of the pubian symphysis. Average carcass backfat thickness (CBF) was computed as the mean of carcass fat depths at the shoulder, the last rib, and the hip joint. Carcasses with head and feet but without kidney fat were then chilled in a cooling room at 4°C for 24 hours. Dressing percentage (DP) was defined as the ratio of cold carcass weight to slaughter weight measured after the fasting period prior to transportation. The day after slaughter, the right half-carcass (RHC) (without head) was weighed and then cut based on a standardized procedure [18]. The ham, loin, belly, shoulder and backfat were weighed separately (HAM, LOIN, BELLY, SHOULDER and BACKFAT, respectively). Lean meat content was then estimated using the cut weights as ELMC (%) = 25.08 – (1.23 × (100 × BACKFAT/RHC)) + (0.87 × (100 × LOIN/RHC)) + (0.73 × (100 × (100 × HAM/RHC)) [19].

Several meat quality traits were recorded on the day after slaughter. Ultimate pH was measured on the *semimembranosus* (pH24 SM) muscle at 4°C using a Knick Portaness 910 pH meter (Knick GmbH & Co., Berlin, Germany) equipped with a Mettler Toledo Probe (Mettler-Toledo International Inc., Urdorf, Switzerland). Meat color was assessed on the *gluteus superficialis* (GS) muscle using the three coordinates, L*, a* and b*, of the CIELAB color space with a Minolta CR-300 chromameter (Konica Minolta, Tokyo, Japan) under the D65 illuminant option and an 11-mm orifice (L*GS, a*GS and b*GS). Under this system, L* indicates how light the meat color is (a lower value being associated with darker meat) while a* represents the degree of green-redness (redder meat for a higher a* value) and b* reflects the degree of blue-yellowness of the meat (yellower meat for a greater b* value).

The water holding capacity (WHC) of GS was measured as the time necessary for a piece of filter paper (about 1 cm^2^) to become wet when placed on the freshly cut surface of the muscle [20], a higher value being associated with a lower ability to lose water (1 point = 10 s; maximum 20 points). A meat quality index (MQI) was computed as a linear combination of pH24 SM, WHC GS and L* GS: MQI (%) = 34 + (11.04 × pH24 SM) + (0.105 × WHC GS) – (0.231 × L* GS). This equation has been defined as a predictor of technological yield (ratio of the weight of cooked ham to the weight of defatted and boneless fresh ham) during cured-cooked ham processing [21].

### Genotyping and genotype quality control

Blood was sampled from the test pigs at a live weight of about 60 kg. The DNA was extracted from blood samples of 491 animals (385 piglets and their 106 sires) and genotyped using the Illumina PorcineSNP60 BeadChip (San Diego, CA, USA) containing 62 163 SNPs [5] at the Centre National de Génotypage (Evry, France). The order of the SNPs was based on the Pig Sscrofa10.2 assembly released by the International Swine Genome Sequencing Consortium [22], combined with RH mapping information [23]. Quality control was done considering genotyping of the 106 sires that were more representative of the LW population. The *check.marker* function of the GenABEL R package [24] was applied. It excluded 5390 SNPs with call rates lower than 97%, 12 077 SNPs with minor allele frequencies less than 5%, and 1051 SNPs with a *P*-value of a *χ*^2^ test for a Hardy-Weinberg equilibrium lower than 1.10^-5^. After applying these quality control measures, 42 272 SNPs located on autosomes and 2140 SNPs that were not located on the Pig Sscrofa 10.2 assembly were retained for association analyses, i.e. an average of 15 SNPs per Mb. After filtering, the number of SNPs per chromosome ranged from 1047 (SSC18 i.e. *Sus scrofa* chromosome 18) to 5155 (SSC1). The call rate across the retained SNPs was higher than 90% for all animals and so all animals were retained for analyses.

### Statistical analyses

#### ***Adjustment of data for systematic environmental effects***

Prior to GWAS, the phenotypes of the 385 LW animals were analyzed jointly with the phenotypes of their batch mates (3030 animals from five breeds) to adjust the data for systematic environmental effects. All traits were corrected using a linear model (GLM procedure, SAS Inst., Inc., Cary, NC [25]), which included the fixed effects of breed (five levels), the combination of test year, test station and contemporary group (slaughter date for meat quality traits or fattening batch for other traits; 20 and 97 levels, respectively) and body weight of the animal at the start of the test (for traits recorded during growth) or at slaughter (for traits recorded at the abattoir), nested within breed, as a linear covariate. The residuals from these analyses were then used as trait phenotypes for GWAS.

#### ***Genome-wide association studies***

The GWAS were performed using the GenABEL R package [24]. For each trait, SNP effects were tested with the FASTA (FAmily-based Score Test for Association) method [26] based on a mixed animal model (1) that included the genomic kinship matrix **G** (*ibs* procedure in GenABEL) to account for relatedness in the sampled population:

(1) $$Y_{j}=\mu +b_{\mathrm{ij}}M_{i}+u_{j}+e_{\mathrm{ij}}$$

with *Y*_*j*_ = the phenotype corrected for systematic environmental effects; *μ* = the overall mean; *b*_*ij*_ = the genotype score (0, 1 or 2) of the *i*^th^ SNP for the *j*^th^ individual; *M*_*i*_ = the additive effect of the *i*^th^ SNP; *u*_*j*_ = the random polygenic effect of the *j*^th^ individual, with covariance structure $u_{j}∼N\left(0,G\sigma_{u}^{2}\right)$, where **G** is the genomic kinship matrix and $\sigma_{u}^{2}$ is the polygenic variance; and *e*_*ij*_ = the random residual effect with $e_{\mathrm{ij}}∼N\left(0,I\sigma_{e}^{2}\right)$, where **I** is an identity matrix and $\sigma_{e}^{2}$ is the residual variance. As a first step, the variance components $\sigma_{u}^{2}$ and $\sigma_{e}^{2}$ were estimated using the genomic kinship matrix in an animal mixed model without a marker effect. These variance components were used in a second step to jointly estimate all the effects included in model (1).

To avoid inflation in the test statistic due to potential deviations from its assumptions, the consistency with *χ*^2^ tests for the distribution of the *P*-values was controlled by regressing the observed *P*-values of each GWAS against the expected *P*-values of a *χ*^2^ test. The *P*-values were then corrected by multiplying observed *P*-values by the regression factor λ, using the genomic control method [27]. This correction assumes that the number of SNPs with an effect on the trait is very small compared to the total number of SNPs tested.

As proposed in Teyssèdre et al. [28], three *P*-value thresholds were used to identify and describe regions of interest. The most stringent threshold was 5.10^-6^, which corresponds to approximately 10 000 independent tests corrected with Bonferroni. A less stringent threshold of 5.10^-5^ was also applied to detect moderate associations, as proposed by the Wellcome Trust Case Control Consortium [29]. Finally, in order to take into account effects of QTL on correlated traits, SNPs with a *P*-value threshold of 5.10^-4^ that were located in the vicinity of the QTL regions were also reported. A QTL region was considered by grouping SNPs with *P*-values lower than 5.10^-4^ in a 10 Mb interval.

#### ***Haplotype blocks and multiple regression analyses***

Haplotypes transmitted by a parent to each of its offspring were inferred based on informative SNPs, using a similar procedure to that of Coop et al. [30] and described in Tortereau et al. [31]. Briefly, the haplotype reconstruction procedure is based on three steps. First, within each half-sib family, a partial haplotype phase was reconstructed for the father based on the genotypes of the offspring. This was done by first partially reconstructing the haplotype transmitted by the father based on markers that are homozygous in the offspring. Then, paternally transmitted haplotypes of all offspring were combined to reconstruct the haplotypes of the father and the haplotype transmitted to each offspring by its mother. Second, the model of Scheet and Stephens [32] was fitted to the partial haplotypes of fathers and mothers across families to increase the level of haplotype reconstruction. Finally, given the phase information in the fathers and for each offspring, segregation indicators that describe which of the paternal alleles was transmitted at each SNP, were reconstructed.

In regions that contained at least one SNP with significant effects on a trait (*P*-value < 5.10^-6^), the LD between SNPs was calculated as *r*^*2*^, using Haploview (V4.2; [33]). LD blocks were generated for SNPs that were separated by less than 500 kb, as proposed by Gabriel et al. [34]. In regions that contained at most 10 SNPs, the haplotypes of the progeny were inferred from their genotypes and phased genotypes of their parents and additional haplotype analyses were performed using the following multiple regression mixed model for each region:

(2) $$Y_{\text{ijk}}=\mu +\sum_{i=1}^{t}\beta_{\mathrm{ij}}H_{i}+S_{j}+e_{\text{ijk}}$$

where *Y*_*ijk*_ = the phenotype corrected for systematic environmental effects; *μ* = the overall mean; *β*_*ij*_ = the haplotype score (0, 1 or 2) of the *i*^th^ haplotype for the *j*^th^ individual, with *t* = the number of haplotypes segregating in the population for that region; *H*_*i*_ = the effect of the *i*^th^ haplotype; *S*_*j*_ = the random sire effect and *e*_*ijk*_ = the random residual effect. Model (2) was tested using the Mixed procedure of the SAS software (SAS Inst., Inc., Cary, NC [25]). The overall effect of the haplotypes in a region and contrasts between two haplotype effects were tested using the CONTRAST and ESTIMATE functions, respectively.

## Results

**Figure 1:**
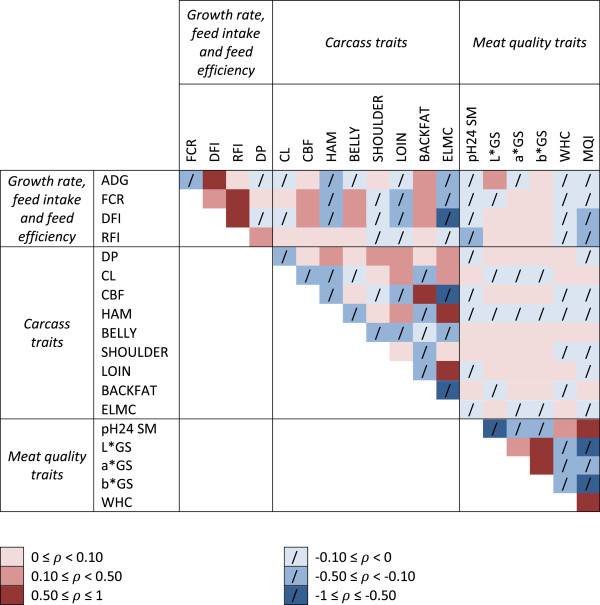
**Magnitude of phenotypic correlations (*ρ*) between traits analysed.** Correlations were estimated on phenotypes adjusted for systematic environmental traits and negative and positive correlations are represented in blue and red, respectively.

For the 19 traits analyzed, the number of records, means and standard deviations are in Table 1. Phenotypic correlations between traits corrected for systematic environmental effects are in Figure 1.

**Table 1:** Descriptive statistics and abbreviations for the traits analyzed

**Trait**	**Abbreviation**	**N**	**Mean**	**STD**
**Growth, feed intake and feed efficiency**				
Average daily gain during the test period (kg.d^-1^)	ADG	385	0.974	0.089
Daily feed intake (kg.d^-1^)	DFI	385	2.61	0.25
Residual feed intake (kg.d^-1^)	RFI	385	0.00	0.13
Feed conversion ratio (kg.kg^-1^BW)	FCR	385	2.68	0.21
**Carcass traits**				
Dressing percentage (%)	DP	385	78.7	1.4
Carcass length (mm)	CL	385	1010	29
Mean carcass backfat thickness (mm)	CBF	385	23.5	3.2
Ham weight (kg)	HAM	385	9.71	0.65
Belly weight (kg)	BELLY	385	5.14	0.53
Shoulder weight (kg)	SHOULDER	385	9.46	0.66
Loin weight (kg)	LOIN	385	10.90	0.88
Backfat weight (kg)	BACKFAT	385	3.42	0.65
Lean meat content calculated with cut weights (%)	ELMC	385	56.0	3.1
**Meat quality traits**				
Ultimate pH of *semimembranosus* muscle	pH24 SM	385	5.70	0.17
L* of *gluteus superficialis* muscle	L*GS	384	50.9	4.0
a* of *gluteus superficialis* muscle	a*GS	384	9.4	1.6
b* of *gluteus superficialis* muscle	b*GS	384	5.5	1.8
Water holding capacity of *gluteus superficialis* muscle (10s)	WHC	385	13.5	6.3
Meat Quality Index	MQI	384	86.9	2.8

N = number of records, STD = phenotypic standard deviation.

### Genome-wide association studies

After checking that no structure was present in our population by applying multidimensional scaling available in the GenABEL R package [24], GWAS analyses were performed for the 19 traits. The average inflation factor of *P*-values was 1.11 ± 0.12, with a minimum of 1 (for five of the 19 traits) and a maximum of 1.39 (for BACKFAT), indicating relatively good concordance between the observed and assumed distributions of the test statistics.

**Figure 2:**
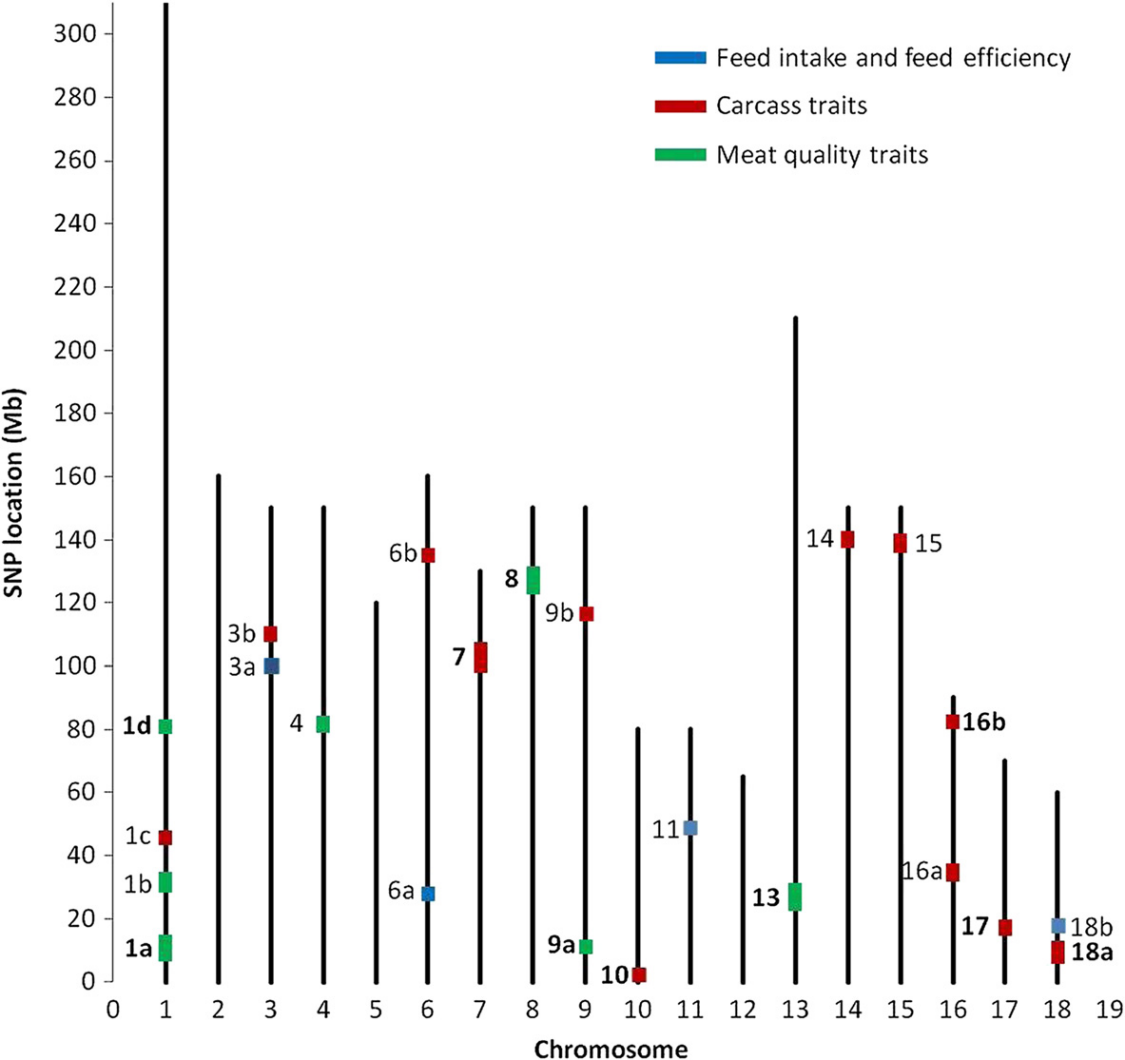
**Distribution of SNPs with *P*-values <5.0E-05 for each group of traits.** The letters distinguish different QTL regions on the same chromosome; when at least one SNP in the region was significant at the threshold of 5.10^-6^, names of the QTL regions are in bold (see Table 2).

Seventeen trait × SNP tests, involving 16 distinct SNPs, were significant at the threshold of 5.10^-6^ and 52 tests, involving 48 distinct SNPs, were significant at the threshold of 5.10^-5^. Only two of these SNPs were not located on the Pig Sscrofa10.2 draft. In total, 23 QTL regions were identified, including all autosomes except SSC2, 5 and 12 (Figure 2). At least one QTL region was identified for each trait, except ADG, DFI, SHOULDER and WHC. The magnitude of the estimated SNP effects was expressed in trait phenotypic standard deviation (STD) units, which was calculated based on phenotypes adjusted for systematic environmental effects (Table 2).

**Table 2:** **QTL regions with at least one SNP with a *P*-value less than 5.10**^**-5**^

	**QTL**^**1**^	**Thresholds**^**2**^			**Positions of flanking markers (kb)**	**Traits**^**3**^	**Effects in STD**^**4 **^**(min-max)**
		**5.10**^**-4**^	**5.10**^**-5**^	**5.10**^**-6**^			
*Growth rate, feed intake, feed efficiency*	3a	2	1	0	100 098–100 119	FCR	0.29 – 0.33
	6a	4	3	0	27 650–28 138	RFI	0.41 – 0.46
	11	1	1	0	48 555	FCR	0.36
	18b	4	1	0	17 724–17 817	FCR	0.29 – 0.33
*Carcass traits*	1c	4	4	0	45 536–45 998	BELLY	0.57 – 0.59
	3b	5	3	0	109 719–110 511	BELLY	0.43 – 0.55
	6b	7	1	0	134 691–135 078	BACKFAT- ELMC- HAM	0.25 – 0.32
	**7**	**10**	**2**	**1**	**100 145–105 315**	**CL**	**0.28 – 0.40**
	9b	2	1	0	116 328–116 390	LOIN	0.33 – 0.42
	**10**	**3**	**1**	**1**	**2 015 – 2 080**	**BELLY**	**0.28 – 0.35**
	14	5	1	0	139 323–140 810	BACKFAT- ELMC - CBF - CL	0.29 – 0.39
	15	3	1	0	137 725–139 857	DP	0.30 – 0.52
	16a	6	2	0	34 003–35 190	HAM	0.30 – 0.39
	**16b**	**17**	**5**	**1**	**82 092–82 664**	**BACKFAT- ELMC**	**0.30 – 0.45**
	**17**	**4**	**3**	**3**	**16 788–17 549**	**CL**	**0.28 – 0.42**
	**18a**	**9**	**1**	**1**	**7 813 – 11 122**	**HAM- ELMC - CBF - BELLY**	**0.27 – 0.40**
*Meat quality traits*	**1a**	**14**	**3**	**1**	**8 715–12 869**	a*GS - b*GS - L*GS	**0.27 – 0.35**
	1b	7	2	0	30 337–32 684	pH24 SM - b*GS - MQI	0.28 – 0.32
	**1d**	**16**	**8**	**4**	**80 701–80 884**	b*GS - L*GS - MQI	**0.30 – 0.49**
	4	5	2	0	80 963–82 252	a*GS	0.27 – 0.38
	**8**	**6**	**2**	**2**	**124 786–129 300**	pH24 SM - b*GS - MQI	**0.29 – 0.45**
	**9a**	**7**	**3**	**2**	**11 043–11 445**	L*GS - MQI	**0.27 – 0.37**
	**13**	**3**	**1**	**1**	**24 391–29 002**	L*GS - MQI	**0.29 – 0.75**

Lines in bold are regions where at least one SNP had a *P*-value < 5.10^-6^; ^1^QTL region as defined by the chromosome number and location (letters are ordered by positions on chromosomes, see Figure 2); ^2^For each QTL region, the number of SNP × trait combinations with a *P*-value lower than the corresponding threshold; ^3^see Table 1 for trait abbreviations; ^4^STD = phenotypic standard deviation estimated on phenotypes adjusted for systematic environmental effects.

Ten QTL regions were identified at the most stringent threshold (*P*-value < 5.10^-6^), i.e. five for carcass traits, five for meat quality traits and none for growth rate, feed intake and feed efficiency. Two of the five carcass QTL, on SSC7 and SSC17, had an effect on carcass length (from 0.28 to 0.42 STD) only. The three other carcass QTL, on SSC10, 16 and 18, affected carcass cut weight and backfat thickness (0.27 - 0.45 STD). The five QTL for meat quality traits were located on SSC1 (two QTL), SSC8, 9 and 13. Each of these QTL affected several meat quality traits, with effects ranging from 0.27 to 0.75 STD. Most of the detected QTL regions were composed of a single SNP that was significant at the 5.10^-6^ threshold, except for the QTL region on SSC17 for CL (three SNPs), and on SSC1d (four SNP) and SSC9a (two SNP) for meat quality traits. However, for each of the 10 most significant QTL regions, other trait × SNP combinations were found at the 5.10^-5^ and 5.10^-4^ thresholds.

Based on the moderate threshold (*P*-value < 5.10^-5^), 13 additional QTL regions were identified. Three QTL, on SSC3, SSC11 and SSC18, had effects of about 0.30 STD on FCR, and one QTL on SSC6 had effects ranging from 0.41 to 0.46 STD for RFI. Seven QTL with effects on different carcass composition traits (0.25 – 0.59 STD) were detected on SSC1, 3, 6, 9, 14, 15 and 16. For meat quality traits, two additional QTL regions (on SSC1b and SSC4) were identified, with moderate effects (0.27 - 0.38) on meat color and ultimate pH.

**Figure 3:**
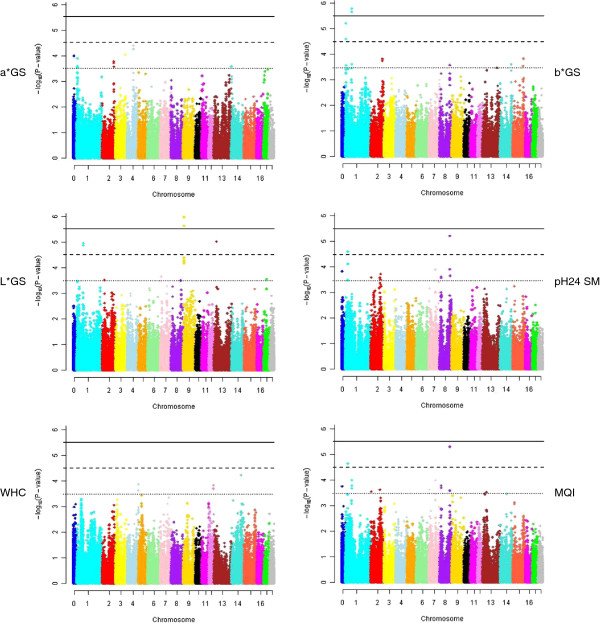
**−log**_**10**_**(*P*-value) of the SNPs tested for meat quality traits plotted against their positions.** See Table 1 for meat quality trait abbreviations; 42 272 SNPs located on autosomes 1 to 18, and 2140 SNPs that are not located (chromosome noted 0) on the *Sus Scrofa* build 10.2 represented by different colors; dotted, dashed and solid lines correspond to thresholds of 5.10^-4^, 5.10^-5^ and 5.10^-6^, respectively.

Relative to the number of traits analyzed, a larger number of tests with a *P*-value < 5.10^-5^ was obtained for meat quality traits (on average 3.5 significant tests per trait) than for growth rate and feed intake (1.5 significant tests per trait) and for carcass traits (2.8 significant tests per trait). With the criteria that were used to define QTL regions, no QTL region was shown to simultaneously affect different groups of traits (growth, feed intake and feed efficiency, carcass and meat quality traits). However, within meat quality traits for example, some peaks tended to be shared, as for pH24 SM and MQI on SSC8, or for b* GS and L* GS on SSC1 (Figure 3).

### Haplotype analyses

**Figure 4:**
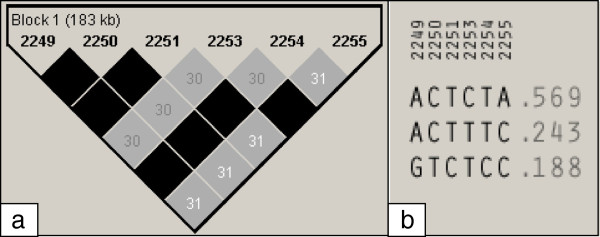
**Haploview plot of linkage disequilibrium (*r***^***2***^**) between significant SNPs on chromosome 1. a**. A black diamond without a number represents complete linkage disequilibrium between the SNP (r^2^ = 1). **b**. Parental frequencies of each haplotype containing the six significant SNPs.

The 10 most significant QTL regions were subjected to haplotype analyses. First, SNPs that had significant effects at the threshold of 5.10^-5^ and that were in high LD within a QTL region were grouped together in haplotype blocks according to the criteria specified by Gabriel et al. [34]. Using these criteria, a haplotype block could be identified for only one of the QTL regions, *i.e.* the 183 kb SSC1d region, with six SNPs that were associated with meat quality traits (Table 2). The *r*^*2*^ between SNPs in this region ranged from 0.30 to 1 (Figure 4a).

Three distinct haplotypes (ACTCTA, ACTTTC and GTCTCC, referred to as Haplo1, Haplo2 and Haplo3 hereinafter) were identified in the SSC1d region with frequencies of 57%, 24% and 19%, respectively (Figure 4b). Their effects on the 19 traits were evaluated for the 376 phased progeny in order to evaluate potential pleiotropic effects of the region. Parental phases were unavailable for nine animals, which were discarded from the haplotype analysis. The haplotypes had significant (0.0001 < *P*-value < 0.01) effects on all six meat quality traits analyzed (Table 3). The highest significance levels were obtained for b*GS and L*GS and for the meat quality index (*P*-value < 1.10^-4^), *i.e.* the traits that displayed significant results in the GWAS (Table 2). For these three traits and for pH24 SM, Haplo1 and Haplo3 showed significant and opposite effects. Haplo1 was associated with favorable effects (e.g. for MQI: +0.5 point for Haplo1 vs -0.6 for Haplo3). Haplo2 had effects that were similar to Haplo1 for b*GS and L*GS, but had no significant effect on MQI and pH24 SM. For the two other traits, Haplo1 was the only haplotype that had a significant effect on a*GS, while Haplo1 and Haplo2 had, respectively, favorable (+8 s) and unfavorable (-8 s) effects on WHC. As a consequence, Haplo1 had a consistently favorable effect on all meat quality traits recorded, and Haplo3 displayed an almost systematically opposite effect on these traits. The effects of Haplo2 were either intermediate or not significantly different from Haplo1.

**Table 3:** Haplotype effects of the SSC1d QTL for meat quality traits (N = 376 pigs)

**Trait**^**1**^	**STD**	***P*-value**^**2**^	**Haplo1 *ACTCTA***		**Haplo2 *ACTTTC***		**Haplo3 *GTCTCC***	
			**β**^**3**^	***P*-value**^**4**^	**β**^**3**^	***P*-value**^**4**^	**β**^**3**^	***P*-value**^**4**^
pH24 SM	0.15	0.0007	0.16^a^	0.0010	0.03^a^	0.6438	-0.19^b^	0.0021
L*GS	3.49	< 0.0001	-0.16^a^	0.0008	-0.14^a^	0.0282	0.30^b^	< 0.0001
a*GS	1.54	0.0252	-0.13^a^	0.0075	0.04^ab^	0.4994	0.09^b^	0.1508
b*GS	1.43	< 0.0001	-0.18^a^	0.0002	-0.13^a^	0.0505	0.31^b^	< 0.0001
WHC (s)	59.6	0.0113	0.14^a^	0.0042	-0.13^b^	0.0472	-0.02^ab^	0.80
MQI (%)	2.45	< 0.0001	0.20^a^	< 0.0001	0.03^a^	0.5838	-0.23^b^	0.0002

^1^See Table 1 for trait abbreviations; ^2^*P*-value of the haplotype effect in a linear mixed model including a mean, a random sire effect and multiple regression on the haplotypes; ^3^estimates of regression coefficients (β) in phenotypic standard deviation (STD) units of the traits estimated using phenotypes adjusted for systematic environmental traits; for a given trait, values with different superscripts (^a^ or^b^) were significantly different (P < 0.05); ^4^*P*-values for the test of β ≠ 0.

## Discussion

The PorcineSNP60 BeadChip has become available quite recently [5]. A GWAS method testing individual SNP effects was chosen because previous studies had demonstrated that single-marker tests produced similar or even greater power than haplotype-based approaches [35,36]. Moreover, testing individual SNP effects does not depend on SNP positions and haplotype reconstruction. None of the detected QTL regions displayed significant effects on more than one group of traits, despite the presence of significant genetic correlations [17], such as between growth rate and body composition. During preliminary simulation studies, the relatively limited size of the data set (about three half-sib progeny from about 100 sires) was shown to enable detection of only relatively large QTL (with effects greater than 0.5 STD) for traits with a heritability of 0.5, and power was less than 30% when the effect of the QTL or trait heritability was reduced [37]. With such limited power, it is therefore not surprising that only large QTL were detected, and that no region was identified to affect multiple traits with moderate genetic correlations. Nevertheless, a relatively large number of significant QTL was found for meat quality traits, although these traits are generally less heritable than growth, feed intake or carcass traits [17].

LD blocks were used to identify haplotypes associated with phenotypes in our study. Only one region could be dissected based on this approach, since the other regions displayed no LD blocks for the SNPs with significant effects. This small number of LD blocks might be due to the distance between SNPs with significant effects or to local inaccuracies in the published sequence or to the presence of limited LD between adjacent SNPs in our population. However, in QTL regions for which LD blocks were identified, it was possible to identify haplotypes that significantly affected the traits.

Alignment of the genetic and physical maps on the most recent porcine genome sequence assembly (Sscrofa10.2) in PigQTLdB [1] allowed our results to be compared with previously reported QTL locations. Ten of the QTL regions found in our study were consistent with QTL locations previously detected by linkage analyses. However, most of the QTL regions detected in our study (13 of the 23 QTL regions) were not previously described. In particular, none of the regions detected coincided with the QTL regions reported by Tribout et al. [38], who used a grand-daughter design with microsatellite markers in the same population. The discrepancies between population structures, methods of analysis, and density and informativity of markers could explain the differences between the results of Tribout et al. [38] and our study.

### QTL for growth rate, feed intake and feed efficiency

Four QTL regions had a significant effect (*P*-value < 5.10^-5^) on RFI (SSC6a) or FCR (SSC3a, 11 and 18b). Markers flanking the SSC6a QTL region are located in the *FTO* gene (*fat mass and obesity associated*), where a polymorphism has previously found to be associated with RFI in Yorkshire pigs [39], but which was subsequently not reported as significantly associated with RFI in a GWAS in that same population [13]). Among the three QTL that affected FCR, two (SSC3a and SSC18b) were not previously reported in the literature. These two QTL regions were small (about 21 and 92 kb respectively) and they had an effect of about 1/3 STD on FCR. In the vicinity of the SSC11 region, a QTL for FCR was previously described in a F2 Meishan × Large White population [40]. Another recent GWAS for FCR in a Duroc population [12] identified no common QTL regions with our LW pigs.

### QTL for carcass traits

Most earlier QTL linkage analyses included carcass traits and a very large number of QTL have been reported for these traits. Nevertheless, to our knowledge, eight of the 12 QTL regions found for carcass traits in our study were not previously described, *i.e.* on SSC1c (at 46 Mb), SSC3b (at 110 Mb), SSC6b (at 135 Mb), SSC9b (at 116 Mb), SSC10 (at 2 Mb), SSC14 (at 139 Mb), SSC15 (at 138 Mb) and SSC16a (at 34 Mb). Belly weight and carcass length displayed the largest number of significant associations (four distinct QTL regions for belly weight and three for carcass length). This might be because these two traits are not included in the French LW breeding objectives, so that QTL that affect these traits are less likely to have been fixed by selection than QTL for traits included in selection. In a purebred commercial population, our study confirmed the presence of four QTL that were previously detected in crossbred populations. Of these, a QTL on SSC7b that influences carcass length was previously described in several crossbred populations [41-43]. Liu et al. [44] also reported a QTL with an effect on backfat thickness in the SSC16b region. The most significant association found in our study (*P*-value = 7.10^-7^), *i.e.* the QTL on SSC17 (at 17 Mb) for carcass length, is located in a region where a QTL was previously described by Karlskov-Mortensen et al. [45] in crossbred Hampshire and Landrace pigs. Finally, in the SSC18 QTL region (at 11 Mb), a QTL was previously described in an F2 Berkshire × Yorkshire population [46].

These results show that several QTL that affect carcass traits with moderate to strong effects (from 0.3 to 0.6 STD) are still segregating in this LW population, despite more than 30 generations of selection for these traits. However, during the past two decades, this population has also been selected for reproductive traits, so that part of the selection pressure is applied to maternal abilities and prolificacy. This may explain why some QTL with relatively strong effects are still segregating in this population, either because selection pressure has not been sufficient to fix them, or because they exert antagonistic effects on production or reproduction traits. As a consequence, although the effects of our QTL must be confirmed in a larger population, estimates of their effects on reproductive traits are also required, in order to decipher how they can actually be used in the context of future marker-assisted selection strategies.

### QTL for meat quality traits

Three of the seven QTL regions detected for meat quality traits were not previously described in the literature: the SSC1a region (at about 8–13 Mb), the SSC9a region (at about 11 Mb) and the SSC13 region (24–29 Mb). The first two QTL regions have moderate effects on meat color traits (about 0.33 STD), while the SSC13 QTL has an effect of 0.75 STD on meat lightness. Three QTL for meat quality traits were previously reported for the SSC1b, SSC4 and SSC8 regions by Ponsuksilii et al. [47] and van Wijk et al. [48] in crossbred populations, while the SSC1d QTL region has been shown to influence meat quality traits in Landrace pigs [49].

In the SSC1d region, a cluster of six SNPs was identified, and the three corresponding haplotypes had significant effects on all meat quality traits analyzed in our study, but not on other production traits. In this region of 183 kb, no functional candidate gene based on the present draft of the pig sequence could be identified. The 106 half-sib families in our study are representative of this LW population at the time of sampling, so that the estimated haplotype frequencies are expected to be close to those in the whole population. Assuming random mating, with the frequency of the favorable haplotype estimated at 57%, only 32% of the animals are expected to carry two copies of the most favorable haplotype. Thus, 68% of the animals are carriers of at least one unfavorable haplotype and almost 4% of the pigs carry two copies of the most unfavorable haplotype. In addition, although meat quality traits had only moderate phenotypic correlations with growth or intake traits in our study, meat quality traits are known to have antagonistic relationships with feed efficiency traits [50]. Such antagonistic effects were, however, not found for these haplotypes or for any of the significant SNPs for meat quality and either FCR or RFI. The lack of adverse influences of the favorable haplotype on correlated production traits and on traits that were not evaluated in this study, such as reproduction traits, will therefore require specific tests and validation before the haplotype is used in selection. Finally, a survey of haplotypes that segregate in other commercial populations (Landrace, Piétrain, Duroc, etc.) and estimation of their effects on meat quality traits, might be necessary to identify the underlying causal polymorphisms.

## Conclusions

This study in a major French commercial pig population confirmed the segregation of several QTL affecting production and meat quality traits. Some of these QTL had not been reported before, while others were detected in crossbred populations using microsatellite markers. These findings demonstrate that using relatively dense SNP arrays within a purebred population makes it possible to detect QTL regions that were not detected by linkage analyses. Given the number of traits analyzed, the largest number of significant associations was obtained for meat quality traits. As selection has until recently tended to focus on growth or carcass traits rather than meat quality, genes with moderate to strong effects are more likely to be still segregating for these traits. However, our results also show that some QTL with moderate to strong effects on feed efficiency and carcass traits continue to segregate in this LW population. Moreover, the QTL detected did not affect multiple types of traits, which suggests that SNP could be used to improve growth, feed intake, feed efficiency and carcass traits without degrading meat quality traits and, reciprocally, to improve meat quality traits without affecting other production traits. This needs further validation to overcome the relatively limited power of our design.

## Competing interests

The authors declare that they have no competing interests.

## Authors’ contributions

MPS performed GWAS and haplotype analyses and drafted the manuscript. TT supervised the performance testing and performed the statistical correction of phenotypes. NI was responsible for blood sampling, DNA extractions and the coordination of genotyping. MB and NM were responsible for performance testing in the experimental unit. BS carried out the parental phase reconstruction. PD was responsible for the genotyping database. MdPS and AT performed the preliminary simulations and GWAS analyses. MJM participated in conducting the experiment, as representative of BIOPORC breeding organizations. HG and JPB supervised the overall analysis and helped to draft the manuscript. DM and CRG had scientific responsibility for the Delisus and Immopig projects, respectively. All authors read and approved the final manuscript.

## References

[B1] Hu Z-L Park CA Wu X-L Reecy JM Animal QTLdb: an improved database tool for livestock animal QTL/association data dissemination in the post-genome era Nucleic Acids Res 2013 41 D871 D879 10.1093/nar/gks1150 23180796 PMC3531174

[B2] Sanchez MP Riquet J Iannuccelli N Gogué J Billon Y Demeure O Caritez JC Burgaud G Fève K Bonnet M Péry C Lagant H Le Roy P Bidanel JP Milan D Effects of quantitative trait loci on chromosomes 1, 2, 4, and 7 on growth, carcass, and meat quality traits in backcross Meishan x Large White pigs J Anim Sci 2006 84 526 537 16478944 10.2527/2006.843526x

[B3] Riquet J Gilbert H Servin B Sanchez M-P Iannuccelli N Billon Y Bidanel J-P Milan D A locally congenic backcross design in pig: a new regional fine QTL mapping approach miming congenic strains used in mouse BMC Genet 2011 12 6 21235745 10.1186/1471-2156-12-6 PMC3748014

[B4] Rothschild M Bidanel JP Current status of quantitative trait locus mapping in pigs Pig News and Information 2002 23 39N 54N

[B5] Ramos AM Crooijmans RPM Affara NA Amaral AJ Archibald AL Beever JE Bendixen C Churcher C Clark R Dehais P Hansen MS Hedegaard J Hu ZL Kerstens HH Law AS Megens HJ Milan D Nonneman DJ Rohrer GA Rothschild MF Smith TPL Schnabel RD Van Tassell CP Taylor JF Wiedmann RT Schook LB Groenen MAM Design of a high density SNP genotyping assay in the pig using SNPs identified and characterized by next generation sequencing technology PLoS One 2009 4 e6524 10.1371/journal.pone.0006524 19654876 PMC2716536

[B6] Kemper KE Daetwyler HD Visscher PM Goddard ME Comparing linkage and association analyses in sheep points to a better way of doing GWAS Genet Res 2012 94 191 203 10.1017/S0016672312000365 22950900

[B7] Duijvesteijn N Knol EF Merks JWM Crooijmans RPMA Groenen MAM Bovenhuis H Harlizius B A genome-wide association study on androstenone levels in pigs reveals a cluster of candidate genes on chromosome 6 BMC Genet 2010 11 42 20487517 10.1186/1471-2156-11-42 PMC2889844

[B8] Onteru SK Fan B Nikkila MT Garrick DJ Stalder KJ Rothschild MF Whole-genome association analyses for lifetime reproductive traits in the pig J Anim Sci 2011 89 988 995 10.2527/jas.2010-3236 21183715

[B9] Fan B Onteru SK Du ZQ Garrick DJ Stalder KJ Rothschild MF Genome-wide association study identifies loci for body composition and structural soundness traits in pigs PLoS One 2011 6 e14726 10.1371/journal.pone.0014726 21383979 PMC3044704

[B10] Fu WX Liu Y Lu X Niu XY Ding XD Liu JF Zhang Q A genome-wide association study identifies two novel promising candidate genes affecting Escherichia coli F4ab/F4ac susceptibility in swine PLoS One 2012 7 e32127 10.1371/journal.pone.0032127 22457712 PMC3311625

[B11] Wang JY Luo YR Fu WX Lu X Zhou JP Ding XD Liu JF Zhang Q Genome-wide association studies for hematological traits in swine Anim Genet 2012 44 34 43 22548415 10.1111/j.1365-2052.2012.02366.x

[B12] Sahana G Kadlecová V Hornshøj H Nielsen B Christensen OF A genome-wide association scan in pig identifies novel regions associated with feed efficiency trait J Anim Sci 2013 91 1041 1050 10.2527/jas.2012-5643 23296815

[B13] Onteru SK Gorbach DM Young JM Garrick DJ Dekkers JCM Rothschild MF Whole genome association studies of residual feed intake and related traits in the pig PLoS One 2013 8 e61756 10.1371/journal.pone.0061756 23840294 PMC3694077

[B14] Becker D Wimmers K Luther H Hofer A Leeb T A genome-wide association study to detect QTL for commercially important traits in Swiss Large White boars PLoS One 2013 8 e55951 10.1371/journal.pone.0055951 23393604 PMC3564845

[B15] Luo W Cheng D Chen S Wang L Li Y Ma X Song X Liu X Li W Liang J Yan H Zhao K Wang C Wang L Zhang L Genome-wide association analysis of meat quality traits in a porcine Large White x Minzhu intercross population Int J Biol Sci 2012 8 580 595 22532790 10.7150/ijbs.3614 PMC3334672

[B16] Luo W Chen S Cheng D Wang L Li Y Ma X Song X Liu X Li W Liang J Yan H Zhao KB Wang CD Wang LX Zhang LC Genome-wide association study of porcine hematological parameters in a Large White x Minzhu F2 resource population Int J Biol Sci 2012 8 870 881 22745577 10.7150/ijbs.4027 PMC3385009

[B17] Saintilan R Mérour I Brossard L Tribout T Dourmad JY Sellier P Bidanel J van Milgen J Gilbert H Genetics of residual feed intake in growing pigs: relationships with production traits, and nitrogen and phosphorus excretion traits J Anim Sci 2013 91 2542 2554 10.2527/jas.2012-5687 23482579

[B18] Métayer A Daumas G Estimation, par découpe, de la teneur en viande maigre des carcasses de porcs Journées de la Recherche Porcine 1998 30 3 26

[B19] Daumas G Taux de muscle des pièces et appréciation de la composition corporelle des carcasses Journées de la Recherche Porcine 2008 40 61 67

[B20] Charpentier J Monin G Ollivier L Correlations between carcass characteristics and meat quality in Large White pigs Proceedings of the 2nd International Symposium on Condition and Meat Quality of Pigs: 22–24 March 1971 1971 Zeist 255 260

[B21] Tribout T Caritez JC Gogué J Gruand J Bouffaud M Le Roy P Bidanel JP Estimation of realised genetic trends in French Large White pigs from 1977 to 1998 for production and quality traits using frozen semen Proceedings of the 54th Annual Meeting of the European Association for Animal Production: August 31 – September 3 2003 2003 Roma paperG4.12

[B22] Groenen MAM Archibald AL Uenishi H Tuggle CK Takeuchi Y Rothschild MF Rogel-Gaillard C Park C Milan D Megens HJ Li ST Larkin DM Kim H Frantz LAF Caccamo M Ahn H Aken BL Anselmo A Anthon C Auvil L Badaoui B Beattie CW Bendixen C Berman D Blecha F Blomberg J Bolund L Bosse M Botti S Zhan BJ Analyses of pig genomes provide insight into porcine demography and evolution Nature 2012 491 393 398 10.1038/nature11622 23151582 PMC3566564

[B23] Servin B Faraut T Iannuccelli N Zelenika D Milan D High-resolution autosomal radiation hybrid maps of the pig genome and their contribution to the genome sequence assembly BMC Genomics 2012 13 585 10.1186/1471-2164-13-585 23153393 PMC3499281

[B24] Aulchenko YS de Koning DJ Haley CS Genomewide rapid association using mixed model and regression: a fast and simple method for genomewide pedigree-based quantitative trait loci association analysis Genetics 2007 177 577 585 10.1534/genetics.107.075614 17660554 PMC2013682

[B25] Institute SAS User's guide: statistics Statistical Analysis Systems Institute Inc 1999 8 Cary, NC

[B26] Chen WM Abecasis GR Family-based association tests for genomewide association scans Am J Hum Genet 2007 81 913 926 10.1086/521580 17924335 PMC2265659

[B27] Devlin B Roeder K Genomic control for association studies Biometrics 1999 55 997 1004 10.1111/j.0006-341X.1999.00997.x 11315092

[B28] Teyssedre S Dupuis M Guérin G Schibler L Denoix JM Elsen JM Ricard A Genome-wide association studies for osteochondrosis in French Trotter horses J Anim Sci 2012 90 45 53 10.2527/jas.2011-4031 21841084

[B29] Burton PR Clayton DG Cardon LR Craddock N Deloukas P Duncanson A Kwiatkowski DP McCarthy MI Ouwehand WH Samani NJ Todd JA Donnelly P Barrett JC Davison D Easton D Evans D Leung HT Marchini JL Morris AP Spencer CCA Tobin MD Attwood AP Boorman JP Cant B Everson U Hussey JM Jolley JD Knight AS Koch K Meech E Genome-wide association study of 14,000 cases of seven common diseases and 3,000 shared controls Nature 2007 447 661 678 10.1038/nature05911 17554300 PMC2719288

[B30] Coop G Wen X Ober C Pritchard JK Przeworski M High-resolution mapping of crossovers reveals extensive variation in fine-scale recombination patterns among humans Science 2008 319 1395 1398 10.1126/science.1151851 18239090

[B31] Tortereau F Servin B Frantz L Megens H-J Milan D Rohrer G Wiedmann R Beever J Archibald A Schook L Groenen M A high density recombination map of the pig reveals a correlation between sex-specific recombination and GC content BMC Genomics 2012 13 586 10.1186/1471-2164-13-586 23152986 PMC3499283

[B32] Scheet P Stephens M A fast and flexible statistical model for large-scale population genotype data: applications to inferring missing genotypes and haplotypic phase Am J Hum Genet 2006 78 629 644 10.1086/502802 16532393 PMC1424677

[B33] Barrett JC Fry B Maller J Daly MJ Haploview: analysis and visualization of LD and haplotype maps Bioinformatics 2005 21 263 265 10.1093/bioinformatics/bth457 15297300

[B34] Gabriel SB Schaffner SF Nguyen H Moore JM Roy J Blumenstiel B Higgins J DeFelice M Lochner A Faggart M Liu-Cordero SN Rotimi C Adeyemo A Cooper R Ward R Lander ES Daly MJ Altshuler D The structure of haplotype blocks in the human genome Science 2002 296 2225 2229 10.1126/science.1069424 12029063

[B35] Grapes L Dekkers JC Rothschild MF Fernando RL Comparing linkage disequilibrium-based methods for fine mapping quantitative trait loci Genetics 2004 166 1561 1570 10.1534/genetics.166.3.1561 15082569 PMC1470790

[B36] Zhao HH Fernando RL Dekkers JC Power and precision of alternate methods for linkage disequilibrium mapping of quantitative trait loci Genetics 2007 175 1975 1986 10.1534/genetics.106.066480 17277369 PMC1855130

[B37] Schneider MP Gilbert H Linkage disequilibrium based methods to map QTL in pig familial populations, a simulation study Proceedings of the 9th World Congress on Genetics Applied to Livestock Production: 1–6 August 2010 2010 Leipzig 4 161

[B38] Tribout T Iannuccelli N Druet T Gilbert H Riquet J Gueblez R Mercat M-J Bidanel J-P Milan D Le Roy P Detection of quantitative trait loci for reproduction and production traits in Large White and French Landrace pig populations Genet Sel Evol 2008 40 61 78 18096115 10.1186/1297-9686-40-1-61 PMC2674919

[B39] Fan B Lkhagvadorj S Cai W Young J Smith RM Dekkers JC Huff-Lonergan E Lonergan SM Rothschild MF Identification of genetic markers associated with residual feed intake and meat quality traits in the pig Meat Sci 2010 84 645 650 10.1016/j.meatsci.2009.10.025 20374837

[B40] Houston RD Haley CS Archibald AL Rance KA A QTL affecting daily feed intake maps to chromosome 2 in pigs Mamm Genome 2005 16 464 470 10.1007/s00335-004-4026-0 16075373

[B41] Nezer C Moreau L Wagenaar D Georges M Results of a whole genome scan targeting QTL for growth and carcass traits in a Pietrain x Large White intercross Genet Sel Evol 2002 34 371 387 10.1186/1297-9686-34-3-371 12081803 PMC2705451

[B42] Sato S Oyamada Y Atsuji K Nade T Kobayashi E Mitsuhashi T Nirasawa K Komatsuda A Saito Y Terai S Hayashi T Sugimoto Y Quantitative trait loci analysis for growth and carcass traits in a Meishan x Duroc F2 resource population J Anim Sci 2003 81 2938 2949 14677848 10.2527/2003.81122938x

[B43] Edwards DB Ernst CW Raney NE Doumit ME Hoge MD Bates RO Quantitative trait locus mapping in an F-2 Duroc x Pietrain resource population: II. Carcass and meat quality traits J Anim Sci 2008 86 254 266 17965326 10.2527/jas.2006-626

[B44] Liu G Kim JJ Jonas E Wimmers K Ponsuksili S Murani E Phatsara C Tholen E Juengst H Tesfaye D Chen JL Schellander K Combined line-cross and half-sib QTL analysis in Duroc-Pietrain population Mamm Genome 2008 19 429 438 10.1007/s00335-008-9132-y 18712441

[B45] Karlskov-Mortensen P Jorgensen CB Fredholm M Identification of 33 microsatellite loci on porcine chromosome 17 Anim Genet 2005 36 258 259 10.1111/j.1365-2052.2005.01269.x 15932410

[B46] Malek M Dekkers JCM Lee HK Baas TJ Rothschild MF A molecular genome scan analysis to identify chromosomal regions influencing economic traits in the pig. I. Growth and body composition Mamm Genome 2001 12 630 636 10.1007/s003350020018 11471058

[B47] Ponsuksili S Chomdej S Murani E Blaser U Schreinemachers HJ Schellander K Wimmers K SNP detection and genetic mapping of porcine genes encoding enzymes in hepatic metabolic pathways and evaluation of linkage with carcass traits Anim Genet 2005 36 477 483 16293120 10.1111/j.1365-2052.2005.01351.x

[B48] van Wijk HJ Buschbell H Dibbits B Liefers SC Harlizius B Heuven HCM Knol EF Bovenhuis H Groenen MAM Variance component analysis of quantitative trait loci for pork carcass composition and meat quality on SSC4 and SSC11 J Anim Sci 2007 85 22 30 10.2527/jas.2006-063 17179536

[B49] Vidal O Noguera JL Amills M Varona L Gil M Jimenez N Davalos G Folch JM Sanchez A Identification of carcass and meat quality quantitative trait loci in a Landrace pig population selected for growth and leanness J Anim Sci 2005 83 293 300 15644499 10.2527/2005.832293x

[B50] Gilbert H Le Roy P Milan D Bidanel JP Linked and pleiotropic QTLs influencing carcass composition traits detected on porcine chromosome 7 Genet Res 2007 89 65 72 10.1017/S0016672307008701 17669227

